# Chikungunya Virus Strains Show Lineage-Specific Variations in Virulence and Cross-Protective Ability in Murine and Nonhuman Primate Models

**DOI:** 10.1128/mBio.02449-17

**Published:** 2018-03-06

**Authors:** Rose M. Langsjoen, Sherry L. Haller, Chad J. Roy, Heather Vinet-Oliphant, Nicholas A. Bergren, Jesse H. Erasmus, Jill A. Livengood, Tim D. Powell, Scott C. Weaver, Shannan L. Rossi

**Affiliations:** a Institute for Translational Sciences, University of Texas Medical Branch, Galveston, Texas, USA; b Institute for Human Infection and Immunity, University of Texas Medical Branch, Galveston, Texas, USA; c Division of Microbiology, Tulane National Primate Research Center, Covington, Louisiana, USA; d Department of Microbiology and Immunology, Tulane School of Medicine, New Orleans, Louisiana, USA; e Division of Veterinary Medicine, Tulane National Primate Research Center, Covington, Louisiana, USA; f Department of Pathology, University of Texas Medical Branch, Galveston, Texas, USA; g Takeda Vaccines, Inc., Fort Collins, Colorado, USA; h Department of Microbiology and Immunology, University of Texas Medical Branch, Galveston, Texas, USA; Vanderbilt University Medical Center

**Keywords:** alphavirus, chikungunya, vaccine

## Abstract

Chikungunya virus (CHIKV) is a reemerging arbovirus capable of causing explosive outbreaks of febrile illness, polyarthritis, and polyarthralgia, inflicting severe morbidity on affected populations. CHIKV can be genetically classified into 3 major lineages: West African (WA); East, Central, and South African (ECSA); Indian Ocean (IOL); and Asian. Additionally, the Indian Ocean (IOL) sublineage emerged within the ECSA clade and the Asian/American sublineage emerged within the Asian clade. While differences in epidemiological and pathological characteristics among outbreaks involving different CHIKV lineages and sublineages have been suggested, few targeted investigations comparing lineage virulence levels have been reported. We compared the virulence levels of CHIKV isolates representing all major lineages and sublineages in the type I interferon receptor-knockout A129 mouse model and found lineage-specific differences in virulence. We also evaluated the cross-protective efficacy of the IOL-derived, live-attenuated vaccine strain CHIKV/IRESv1 against the Asian/American CHIKV isolate YO123223 in both murine and nonhuman primate models, as well as the WA strain SH2830 in a murine model. The CHIKV/IRES vaccine provided protection both in mice and in nonhuman primate cohorts against Caribbean strain challenge and protected mice against WA challenge. Taken together, our data suggest that Asian/American CHIKV strains are less virulent than those in the Asian, ECSA, and WA lineages and that despite differences in virulence, IOL-based vaccine strains offer robust cross-protection against strains from other lineages. Further research is needed to elucidate the genetic basis for variation in CHIKV virulence in the A129 mouse model and to corroborate this variation with human pathogenicity.

## IMPORTANCE

Chikungunya virus (CHIKV) is a reemerging human pathogen capable of causing debilitating and disfiguring polyarthritis, which can last for months to years after initial fever has resolved. There are four major genetic lineages of CHIKV, as well as two recently emerged sublineages, none of which have been evaluated for differences in virulence. Moreover, the ability of chikungunya vaccines to cross-protect against heterologous CHIKV lineages has not been explored. Therefore, we sought to compare the virulence levels among CHIKV lineages, as well as to evaluate the cross-protective efficacy of the CHIKV/IRESv1 vaccine candidate, in two different models of CHIKV infection. Our results suggest that, although significant differences in virulence were observed among CHIKV lineages, the CHIKV/IRESv1 vaccine elicits cross-lineage protective immunity. These findings provide valuable information for predicting the severity of CHIKV-associated morbidity in future outbreaks, as well as vaccine development considerations.

## INTRODUCTION

Chikungunya virus (CHIKV), an important human pathogen in the family *Togaviridae*, genus *Alphavirus*, is the etiologic agent of chikungunya fever (CHIKF), which is marked by a sudden onset of high fever, rash, and polyarthritis/polyarthralgia. This polyarthritis may last months to years after infection, causing significant financial as well as public health burdens. Three major genetically distinct CHIKV lineages have been identified by phylogenetic analyses based on both partial (1) and complete (2, 3) genome sequences: West African (WA); East, Central, and South African (ECSA); and Asian. Another distinct sublineage arising from the ECSA lineage during the Indian Ocean outbreak is called the Indian Ocean lineage (IOL). CHIKV isolates originating from the 2013 Caribbean introduction also form a novel American sublineage within the Asian lineage (Asian/American) (4, 5) (see Fig. S1 in the supplemental material). The most important clades in terms of public health impact are the Asian lineage (including Asian/American) and the IOL sublineage, as well as some other ECSA lineage strains responsible for African outbreaks. These two lineages and sublineages are responsible for multiple CHIKV outbreaks over the last 15 years involving millions of people (3, 4, 6), which continue to arise in Asia and Africa (7–9). Knowledge of WA CHIKV strain outbreaks is limited, particularly in the contemporary literature, which likely reflects the remoteness of the regions where WA strains circulate.

10.1128/mBio.02449-17.1 FIG S1  Evolution of the major chikungunya virus (CHIKV) lineages. (A) Maximum likelihood tree based on chikungunya virus open reading frames with bootstrap values for the most prominent chikungunya virus clades, with strains included in this study highlighted. Download FIG S1, TIF file, 11.3 MB. Copyright © 2018 Langsjoen et al. 2018 Langsjoen et al. This content is distributed under the terms of the Creative Commons Attribution 4.0 International license.

Pathogenesis and virulence variation among CHIKV lineages and sublineages have not been formally investigated, although some comparisons have been made using murine and nonhuman primate (NHP) models. One study comparing virulence levels of Asian lineage and IOL sublineage strains isolated in Malaysia found that, when inoculated into the brains of suckling mice, the Asian strain generated higher mortality and upregulation of proapoptotic genes, whereas the IOL strain caused lower mortality and higher upregulation of antiviral and antiapoptotic genes (10). Conversely, when the IOL sublineage isolate LR2006 OPY1 (LR) and the Caribbean (Asian/American sublineage) isolate CNR20235 were inoculated into adult immunocompetent mice, LR-infected mice showed greater joint inflammation, while CNR20235-inoculated mice showed less evidence of pathological lesions and lower levels of inflammatory cytokines. This study also found differing natural killer cell responses to infection with these two CHIKV strains (11). However, because only two isolates were evaluated in these studies, it is difficult to conclude whether these findings represent strain- or lineage-dependent phenomena. When pregnant rhesus macaques were infected with either a WA or an ECSA strain of CHIKV, no pathophysiological differences (e.g., severity and duration of joint swelling or histopathological observations) were noted, although the WA strain induced a modestly, though not significantly, higher viremia (12). Finally, when Gardner et al. (13) compared infections of C57BL/6 mice with either LR or a 1960s Asian isolate closely related to the AF15561 strain, they found notably less inflammatory infiltrate in the feet of Asian strain-infected mice, as well as lower peak footpad swelling and significantly lower levels of monocyte chemoattractant protein 1 (MCP-1), gamma interferon (IFN-γ), and alpha/beta interferon (IFN-α/β).

In addition to relative strain virulence, the ability of CHIKV strains to cross-protect across heterologous genotypes is important for understanding epidemiology and future risk of outbreaks, as well as for predicting vaccine efficacy. Most evidence of cross-protection has been shown indirectly through evaluation of the neutralizing antibody responses (1). For example, plaque reduction neutralization tests (PRNTs) demonstrating the antibody response to a measles virus-vectored CHIKV vaccine expressing the structural polyprotein of an IOL isolate (14) (MV-CHIK) utilized the Asian lineage-derived 181/clone 25 vaccine strain (15). Murine studies with the 181/clone 25 vaccine also showed cross-neutralizing immunity against ECSA strains (14). Similarly, a phase 1 trial evaluating the VRC-CHIKVLP059-00-VP virus-like particle vaccine expressing structural proteins of the WA CHIKV strain 37997 also used the Asian 181/clone 25 virus strain to demonstrate neutralizing antibody responses, providing further evidence of heterologous lineage cross-neutralization (16). Further study of sera from individuals vaccinated with this strain showed that VRC-CHIKVLP059-00-VP elicited neutralizing antibodies to 9 more CHIKV strains representing the 3 major lineages, although neutralizing capabilities varied significantly between strains (17).

However, recent findings suggest that neutralizing ability of antibodies to heterologous CHIKV strains can also vary significantly. Chua et al. (18) found that immune sera from patients previously infected with either ECSA or Asian CHIKV strains showed differential binding and cross-neutralization efficiency. Although studies specifically evaluating the ability of CHIKV vaccines to protect against challenge from heterologous CHIKV strains are limited, the IOL-based, live-attenuated CHIKV/IRESv1 vaccine protects A129 mice from challenge with the closely related alphavirus O’nyong-nyong virus (ONNV), showing cross-species protection (19). Furthermore, when C57BL/6 mice were vaccinated with a killed Asian CHIKV isolate, animals were fully protected from viremia and footpad swelling post-LR challenge (13); this same study also found that mice previously infected with the closely related arthritogenic alphavirus Ross River virus produced CHIKV-cross-reactive antibodies and were fully protected from CHIKV challenge, again demonstrating cross-species protection.

With the arrival of both ECSA and Asian lineage CHIKV strains in the Americas, it is critical to assess their relative virulence as well as the ability of vaccines to cross-protect. Here, we assessed the virulence of CHIKV isolates derived from 5 different lineages, WA, ECSA, IOL, Asian, and Asian/American, in the lethal type I interferon-deficient A129 mouse model. We also evaluated the ability of the IOL-based CHIKV/IRESv1 vaccine to protect against challenge with an Asian/American CHIKV isolate in both murine and cynomolgus macaque models.

## RESULTS

### Different CHIKV lineages produce differential mortality rates in A129 mice.

The *IFNaR*^*−/−*^ mouse (A129) is useful for evaluating potential differences in CHIKV virulence, as the deficiency in type I interferon signaling results in a lethal outcome, offering the opportunity to compare survival times. Mixed-sex A129 mice, aged 4 to 8 weeks, were inoculated in the left rear footpad with 10 µl of phosphate-buffered saline (PBS) containing approximately 10^4^ PFU of various CHIKV isolates (*n* = 5 to 10 per isolate). Mice were monitored daily for signs of disease, and any that met designated criteria were humanely euthanized with deaths recorded as the following day. Blood was collected from the retroorbital sinus on days 1, 2, and 3 from alternating halves of each cohort.

**FIG 1:**
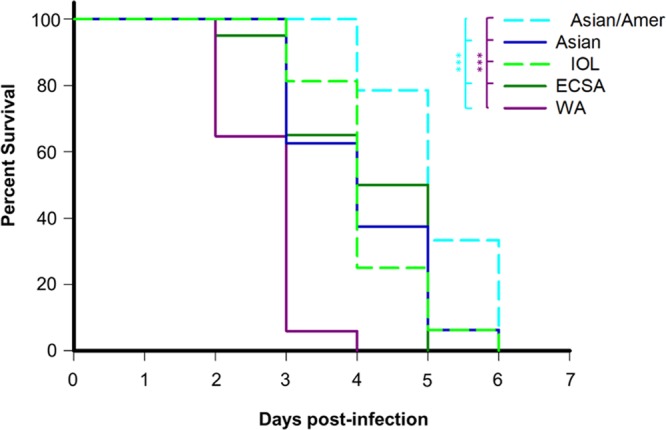
Survival of CHIKV-infected A129 mice differs by lineage. Four- to 8-week-old A129 mice were infected with approximately 10^4^ PFU of a CHIKV isolate from one of 5 genetic CHIKV lineages (*n* = 5 or 6 per group), and survival was assessed, with euthanasia counting as a death on the following day. Isolates were pooled into their respective groups, and survival curves were analyzed by Kaplan-Meier survival analysis using log rank test in SigmaPlot with the Holm-Sidak method of multiple pairwise comparisons. WA strains induced a significant leftward shift in the survival curve compared with all other lineages, while Asian/American strain-infected mice survived significantly longer than mice infected with strains from WA, ECSA, and Asian lineages (*P* << 0.005, denoted by **); survival was not significantly different between ECSA-, IOL-, and Asian lineage-infected mice.

At least two representative, low-passage-number CHIKV strains were selected to represent all major lineages (Table 1). Data for individual strains were pooled into their representative lineage for survival, weight loss, and viremia analyses. All mice succumbed to WA strain infection by 4 days postinfection (DPI), while mice infected with IOL and ECSA strains succumbed by days 4 and 5, respectively. Mice infected with Asian lineage isolates all succumbed to infection by day 5, while mice infected with isolates from the American sublineage survived up to day 6. Log rank tests revealed that survival after either WA or Asian/American strain infection was significantly different from that of other groups (log rank analysis of survival, *P* < 0.005 for WA versus IOL/ECSA/Asian/[Asian/American] and Asian/American versus WA/ECSA/Asian) (Fig. 1). No other significant differences were observed within comparisons between Asian, IOL, or ECSA lineage infections, e.g., IOL versus ECSA/Asian. Survival curves for individual strains are found in Fig. S2 in the supplemental material. Several further analyses exploring the effect of age on survival suggest that 4- to 6- and 8-week-old groups were not significantly different (data not shown).

10.1128/mBio.02449-17.2 FIG S2  Survival of CHIKV-infected A129 mice. Four- to 8-week-old A129 mice were infected with approximately 10^4^ PFU of a CHIKV isolate from one of 5 genetic CHIKV lineages (*n* = 5 to 10 per strain/isolate; light blue, American; dark blue, Asian; light green, IOL; dark green, ECSA; purple, WA), and survival was assessed, with euthanasia counting as a death on the following day. Only Asian strains showed significant within-group variation in time-to-death (log rank analysis, *P* = 0.0115). Download FIG S2, TIF file, 0.5 MB. Copyright © 2018 Langsjoen et al. 2018 Langsjoen et al. This content is distributed under the terms of the Creative Commons Attribution 4.0 International license.

**TABLE 1:** CHIKV isolate information^a^

Strain/isolate	Lineage	Yr	Location	Source	Passage history	Back titer (PFU)
YO111213	Asian/Amer	2014	French Guiana	Human	C6/36-2, Vero-2	1.09 × 10^4^
YO123223	Asian/Amer	2014	Guadeloupe	Human	C6/36-2, Vero-2	1.90 × 10^4^
TA0006	Asian/Amer	2015	Mexico	Human	Vero-2	3.00 × 10^4^
HIII0044	Asian/Amer	2015	Mexico	Mosquito	Vero-2	1.55 × 10^5^
R99659	Asian/Amer	2014	British Virgin Islands	Human	C6/36-2, Vero-2	8.50 × 10^3^
15561	Asian	1962	Thailand	Human	Unk, Vero-2	2.50 × 10^4^
SV0444-95	Asian	1995	Thailand	Human	MK2-1, Vero-1, C6/36-1, Vero-1	1.1 × 10^4^
LR	IOL	2006	La Reunion	Human	Vero-3, SM-1, Vero-1	1.40 × 10^4^
SL-CK1	IOL	2007	Sri Lanka	Human	Vero-2, C6/36-1	2.1 × 10^4^
Bianchi	IOL	2007	Italy	Human	Unk, Vero-2	1.6 × 10^4^
CAR 256	ECSA		Central African Republic	Mosquito	SM-1, C6/36-1, Vero-1	3.4 × 10^4^
LSFS	ECSA	1960	Congo	Human	SM-1, Vero-2, C6/36-1	3.4 × 10^4^
Ross	ECSA	1953	Tanzania	Human	SM-16, Vero-2, C6/36-1	3.4 × 10^4^
SAH2123	ECSA	1976	South Africa	Human	Mosquito-1, SM-2, Vero-1, C6/36-1	7.8 × 10^4^
SH2830	WA	1966	Senegal	Human	SM-3, Vero-2, C6/36-1	3.8 × 10^4^
37997	WA	1983	Senegal	Mosquito	Vero-3	5 × 10^4^

a Abbreviations: Amer, American; Unk, unknown.

**FIG 2:**
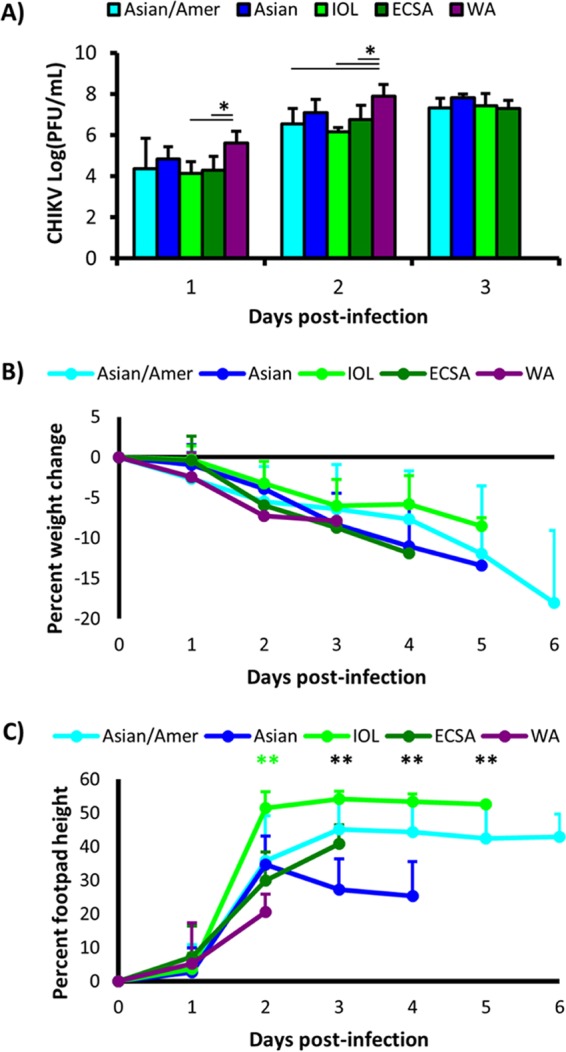
Comparison of viremia, weight, and footpad height changes between A129 mice infected with CHIKV isolates from different lineages. Four- to 8-week-old A129 mice were infected with approximately 10^4^ PFU of a CHIKV isolate from one of 5 genetic CHIKV lineages (*n* = 5 or 6 per strain/isolate), blood was taken on days 1 to 3 from alternating mice to assess viremia (A), and weight (B) and footpad height (C) were monitored daily; data were pooled by lineage for analysis. A129 mice infected with WA CHIKV isolates had significantly higher viremia than those infected with ECSA and IOL isolates 1 day postinfection and those infected with ECSA, IOL, and Asian/American isolates 2 days postinfection (ANOVA, *P* < 0.05 denoted by *). Weight change was not significantly different (one-way ANOVA, *P* = 0.32), while footpad height change was significantly different 2 to 5 DPI (one-way ANOVA, *P* >> 0.05, denoted by **), with IOL strains inducing significantly more footpad swelling on day 2 (one-way ANOVA with Bonferroni *post hoc* test, *P* < 0.01).

WA lineage-infected mice developed significantly higher viremia titers than ECSA- and IOL-infected mice at 1 DPI, and WA strains also produced significantly higher viremia than ECSA, IOL, and Asian/American strains at 2 DPI (analysis of variance [ANOVA], *P* < 0.05) (Fig. 2A). No WA-infected mouse serum was available for plaque assay after 3 DPI, and no other significant differences were observed among any other groups. No significant differences were observed for weight change (one-way ANOVA, *P* = 0.32) (Fig. 2B), while there were significant differences in footpad swelling on days 2 to 6 (one-way ANOVA, *P* >> 0.05) (Fig. 2C); IOL strains induced significantly higher footpad swelling at 2 DPI; however, *post hoc* comparisons were untenable after 2 DPI given the increasingly unequal subject distributions over time.

### **IOL-based CHIKV live-attenuated vaccine protects A129 mice against challenge with CRBN isolate** YO123223.

**FIG 3:**
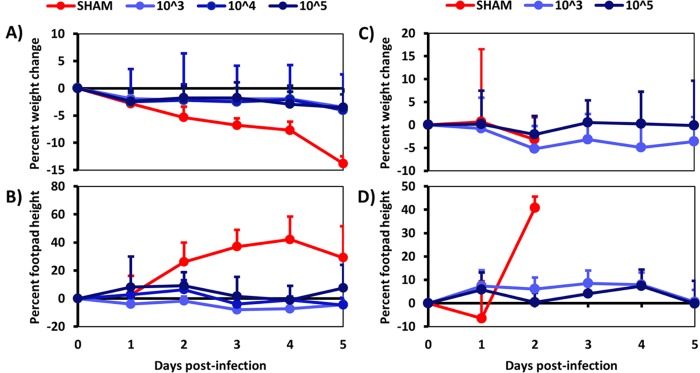
CHIKV vaccine candidate CHIKV/IRESv1 at multiple doses provides complete protection from lethal YO123223 and SH2830 CHIKV challenge in adult A129 mice. (A and B) A129 mice were vaccinated with various doses of CHIKV/IRESv1 vaccine or PBS and then challenged 4 weeks later with Asian/American CHIKV strain YO123223, and weight (A) and footpad height (B) were monitored until all mice in the sham-vaccinated group succumbed to infection. (C and D) A129 mice were vaccinated with either 10^3^ or 10^5^ PFU of CHIKV/IRESv1 vaccine or PBS and then challenged with West African CHIKV strain SH2830, and weight (C) and footpad height (D) were monitored for 5 days.

Previously, we reported the preclinical development and testing of a live-attenuated vaccine that uses a picornavirus internal ribosomal entry site (IRES) to attenuate the LR strain of CHIKV. This vaccine, CHIKV/IRESv1, is highly immunogenic and readily protects both mice and nonhuman primates against challenge with the parental LR strain (20–22). To determine if CHIKV/IRESv1 can protect A129 mice against Asian/American CHIKV isolates, cohorts of 5-week-old A129 mice were vaccinated subcutaneously with 10^3^, 10^4^, or 10^5^ PFU. As previously reported, mice tolerated all vaccine doses well with complete seroconversion. Positive PRNT_80_ titers (>1:20) determined using the Asian/American YO123223 CHIKV strain were observed in all mice at day 14 postvaccination, the earliest time sampled, and continued to rise until challenge (data not shown). Five weeks later, mice were challenged with 10^4^ PFU of YO123223 in the left footpad and then monitored daily as described previously (20). No weight loss or footpad swelling was detected in any vaccinated cohorts, regardless of the CHIKV/IRESv1 dose (Fig. 3A and B), nor was any viremia detected (data not shown). Within 6 days of challenge, all vaccinated mice remained healthy with no significant decline in mean weight, whereas all sham-vaccinated mice lost weight and succumbed to infection. Survivors were held for an additional 2 weeks, and all remained healthy.

Additionally, we vaccinated A129 mice with either PBS or 10^3^ PFU or 10^5^ PFU of CHIKV/IRESv1 and then challenged them with 10^4^ PFU of the WA CHIKV strain SH2830 at 8 weeks postvaccination. All vaccinated mice survived challenge, while sham-vaccinated animals succumbed to infection within 3 days after challenge. No footpad swelling was detected in vaccinated animals, and only insignificant weight loss was observed (Fig. 3C and D). Vaccinated animals were monitored for an additional 2 weeks after challenge, and all remained healthy.

### **IOL-based live-attenuated CHIKV vaccine protects cynomolgus macaques against challenge with American isolate** YO12322**3, which is less pathogenic than historical LR-infected controls.**

**FIG 4:**
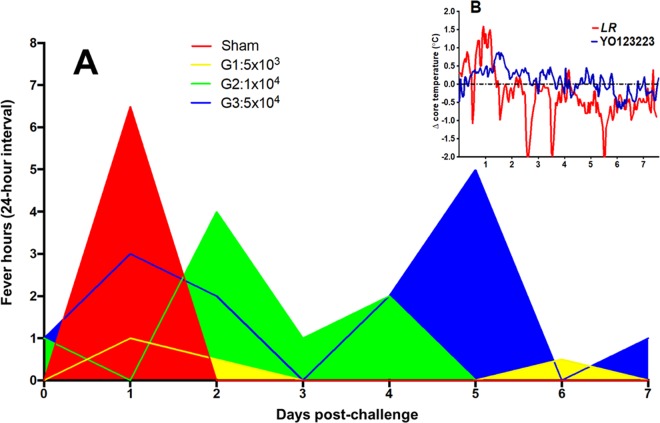
Average fever hours in CHIKV/IRESv1-vaccinated and naive cynomolgus macaques after CHIKV YO123223 challenge. Fever hours are defined as the number of hours within a 24-h time period in which core temperature exceeded 1.5 times the maximum standard deviation of the control temperature value determined prior to challenge. (A) Sham group vaccinated with saline; vaccine dose (in PFU) received by group denoted in color key. (B) Inset graph shows representative sample of core temperature changes (Δ) in sham-vaccinated animals subcutaneously challenged with CHIKV YO123223 or LR2006 OPY1 virus, both at 1.0E+5 PFU. Data were time matched to preexposure values from each animal for derivation of relative temperature change.

**FIG 5:**
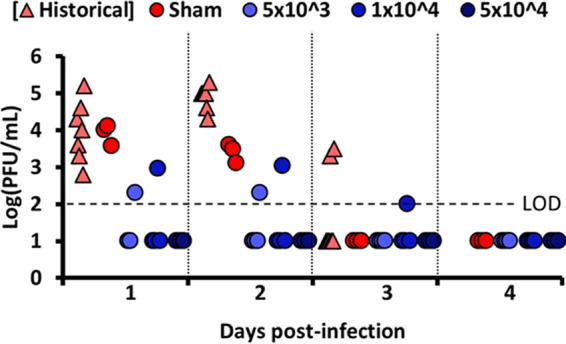
IOL-based CHIKV/IRESv1 CHIKV vaccine prevents viremia in vaccinated cynomolgus macaques challenged with CRBN isolate YO123223. Viremia of cynomolgus macaques vaccinated with CHIKV/IRESv1 prior to viral challenge with CHIKV YO123223 for animals sham vaccinated with saline (red circles; light red triangles indicate historical data for challenge with LR2006) or a prime-only intradermal (100-µl) dose of CHIKV/IRESv1 at either 5.0E+3 (light blue circles), 1.0E+4 (medium blue circles), or 5.0E+4 (dark blue circles) PFU. Dashed line indicates limit of detection (LOD) of plaque assay.

We used age-matched cynomolgus macaques (*Macaca fascicularis*) weighing 3.8 to 7.5 kg, free of simian immunodeficiency virus (SIV), simian type D retrovirus, and simian T-lymphotropic virus and antibodies to western, Venezuelan, and eastern equine encephalitis virus and Sindbis, Semliki Forest, and chikungunya alphaviruses (assayed by hemagglutination inhibition). Prior to vaccination, some animals were surgically implanted with telemetry devices and were allowed to recover for 21 days prior to any manipulation. Baseline physiological data were collected, and each animal’s prevaccination measurements served as control data for comparison with postchallenge values. Macaques were vaccinated with a single intradermal dose of either 5 × 10^3^ (*n* = 4), 1 × 10^4^ (*n* = 4), or 5 × 10^4^ (*n* = 3) PFU of CHIKV/IRESv1 or were sham vaccinated with PBS (*n* = 3). Ten days prior to challenge, serum neutralizing antibody titers were assessed using the LR strain. Seroconversion appeared to be dose dependent, as no animals in the 5 × 10^3^-PFU vaccination group seroconverted (PRNT_80_ titer, >20), and 3 of 4 animals in the 1 × 10^4^-PFU vaccination group seroconverted, while all other CHIKV/IRES-vaccinated animals seroconverted, most with a PRNT_80_ titer of 1:20 and one animal with a titer of 1:160 (data not shown). Seventy days postvaccination, all animals were challenged subcutaneously with 1 × 10^5^ PFU of CHIKV strain YO123223. Animals were monitored daily for signs of clinical illness and telemetrically for 7 days for core body temperature (Fig. 4). Blood was drawn on days 1 to 4 postchallenge to assess viremia (Fig. 5). Additionally, historical data from CHIKV LR challenge of cynomolgus macaques are included in Fig. 4B and 5 (22). Unvaccinated animals exposed to CHIKV strain YO123223 showed minimal signs of illness, including mild anorexia, mild to moderate malaise, and a reduction in activity. These observations appeared to correspond to the level of viremia and small changes in physiological responses, including a 1°C increase of core temperature 30 to 48 h postchallenge. Changes in cardiovascular function, including heart rate, were unremarkable in all animals. No measurable pathological changes, such as joint swelling, were observed upon necropsy, although mild to moderate enlargement of draining (axillary) lymph nodes was noted in two of the four sham-vaccinated animals challenged with Asian/American strain YO123223. Clinical presentation of these sham-vaccinated animals contrasted with that of sham-vaccinated controls challenged previously (22) with IOL strain LR, which included moderate to severe anorexia, extended malaise, and acute (>2.5°C) changes in core temperature, including profound hypothermic events 3 to 6 days postexposure. In the current study, all sham-vaccinated animals developed a viremia postchallenge peaking between 3.5 and 4.1 log_10_ PFU/ml on day 1, and one animal from each of the 5 × 10^3^-PFU- and 1 × 10^4^-PFU-vaccinated groups developed viremia peaking between 2.3 and 3.0 log_10_ PFU/ml, while no animals vaccinated with 5 × 10^4^ PFU developed viremia. Previous LR strain challenge of sham-vaccinated animals resulted in a peak viremia of over 5 log_10_ PFU/ml on day 2, with several animals sustaining viremia through day 3; in contrast, YO123223 challenge resulted in approximately 10-fold-lower viremia titers, which resolved by 3 days postinfection (22).

Subcutaneous exposure to YO123223 in unvaccinated macaques resulted in hyperthermia and a resulting mean 6.5 fever hours approximately 24 h postinoculation (Fig. 4A). In contrast, core body temperatures of CHIKV/IRESv1-vaccinated groups showed mild temperature changes compared to sham-vaccinated animals, ranging from 0 (1 × 10^4^-PFU vaccine dose) to 3 fever hours (5 × 10^4^-PFU vaccine dose) approximately 24 h after YO123223 challenge (Fig. 4A). There were few discernible differences between vaccine dosage groups in mean fever hours. The relatively mild febrile response observed in YO123223-exposed animals is in sharp contrast to the robust pyrexic response and late-stage profound hypothermia observed in LR-exposed macaques in our previous studies (Fig. 4B).

## DISCUSSION

Although multiple lineages are observed, CHIKV is relatively conserved genetically: envelope protein genes can share over 99.8% nucleotide sequence identity among isolates in the same lineage while isolates from different lineages can diverge by 4.4 to 15.5% (23). In amino acid sequence comparisons, the envelope proteins share 95 to 99.9% identity; e.g., IOL strain LR diverges from the Asian/American isolate CNR20235 by 4.49% in the E2 protein (11). On the other hand, even between closely related isolates, nsP3 may differ by more than 6% in amino acid sequences (23). Even though this diversity is relatively limited, particularly in the amino acid sequences, notable differences in pathogenesis and virulence between ECSA and Asian lineage isolates have been reported (10, 11). Furthermore, individual amino acid substitutions, even between closely related ECSA and IOL lineages, can impact *in vitro* measures of virulence, such as plaque size, cytopathic effect, and replication kinetics (24).

Here, we showed that distinct CHIKV lineages vary in virulence for A129 mice. We included WA strains and divided ECSA and Asian strains into relevant sublineages associated with recent epidemics for further analysis. WA strains collectively appeared to be more virulent, with mice universally succumbing to infection within 4 days, while almost 50% of mice survived to day 5 after infection with an Asian/American (Caribbean) strain. Further, WA isolates produced significantly higher levels of viremia than ECSA, IOL, and Asian/American isolates, indicating enhanced replication even in the absence of interferon signaling. Asian/American strains in general appeared to be more attenuated than WA, ECSA, and Asian strains based on these results, although viremia levels were similar. The latter result is not surprising, as Simmons et al. (25) describe high CHIKV levels in blood donations made in Puerto Rico during the CHIKF epidemic of 2014, suggesting that patients were asymptomatic in the face of viremia. Although CHIKV is uniformly lethal in A129 mice, infection with an Asian/American isolate resulted in significantly longer survival than did that with other lineages despite no significant differences in viremia. Additionally, the footpads of infected mice showed significant differences among the various strains, with IOL-infected mice developing significantly higher swelling than all other strains on day 2 postinfection. Footpad data in this study are difficult to interpret, however, for several reasons: footpad measurements were taken by different researchers, introducing potential biases; mice succumbed to infection too quickly to calculate a true peak for some lineages; and CHIKV-induced footpad swelling is due in part to the immune response to infection, which is altered in the A129 model due to the lack of IFN-α/β signaling.

These murine findings suggesting lower virulence levels of Asian/American strains were corroborated by the outcomes of our nonhuman primate (NHP) infections with the Asian/American isolate YO123223. Compared to historical data from animals infected with the IOL strain LR, where fever was followed by pronounced hypothermia (22), we measured no hypothermia in macaques infected with strain YO123223 (22). Also, clinical signs of disease such as malaise and anorexia were markedly milder in YO123223-infected macaques. However, in contrast to our findings in the A129 model indicating no differences in viremia between these isolates, the macaques infected with YO123223 developed a lower-titer viremia than those infected previously with LR (22); the CHIKV strain-dependent difference in viremia induction seen only in the A129 model and not in NHPs may reflect the lack of interferon signaling in the former, which could exaggerate the viremia phenotypes.

The genetic determinants of the apparent differences in virulence that we observed in each animal model among CHIKV lineages are unknown. Although mutations in the envelope glycoprotein genes can have major effects on mosquito infection, none have been identified as a virulence determinant (26). The CHIKV 3′ untranslated genome region (UTR) can influence replication efficiency. For example, IOL strains replicate to higher titers than Asian strains *in vitro* in mosquito cells, and swapping the 3′ UTR demonstrated that this genome region is a major determinant; however, this swap produced effects of different magnitude for each virus (27). Furthermore, a duplication in the 3′ UTR of Asian/American isolates appears to be responsible for enhanced replication in cell cultures (28). Considering the high amino acid identity in the CHIKV proteins but high nucleotide sequence diversity in the untranslated genome regions, UTRs deserve further study. Among the CHIKV strains that we used, the largest difference in virulence was observed between WA lineage and Asian/American sublineage strains, which share an open reading frame nucleotide sequence identity of 84% and a 3′ UTR sequence identity of 51% (see Table S1 in the supplemental material). The low 3′ UTR sequence identity is primarily due to gaps caused by various duplications of several distinct direct repeats rather than point mutations (27, 28), which account for 93% sequence identity in aligned sequences. While it is difficult to predict how sequence changes will affect virulence, studies examining prominent amino acid changes in the coding region of the CHIKV genome, as well as further studies evaluating the effects of UTR swapping between Asian/American and WA CHIKV strains, may identify a genetic basis for these differences in virulence.

10.1128/mBio.02449-17.3 TABLE S1  Sequence identities between CHIKV strain ORFs (nsP1-E1; top, unshaded) and 3′ UTRs (bottom, shaded). Alignments calculated using the EMBOSS-needle pairwise sequence alignment tool (EMBL-EBI; https://www.ebi.ac.uk/Tools/psa/emboss_needle/nucleotide.html) and GenBank sequences. GenBank accession numbers: KX262994.1, KT327167.2, EF452493.1, HM045787.1, DQ443544.2, HM045801.1, HM045793.1, HM045809.1, HM045798.1, and AY726732.1. Download TABLE S1, TIF file, 0.9 MB. Copyright © 2018 Langsjoen et al. 2018 Langsjoen et al. This content is distributed under the terms of the Creative Commons Attribution 4.0 International license.

One limitation inherent to most CHIKV virulence comparisons is that the passage histories of CHIKV strains are not generally uniform. Also, alphaviruses derived from mammalian versus insect cells have different N-linked glycosylation patterns (29) and can also be selected during cell culture passages for the ability to bind glycosaminoglycans (30–34), both potentially affecting their virulence (35–37). The best-known example is the CHIKV vaccine strain 181/clone 25, derived by serially passaging strain 15561 18 times on MRC-5 human lung fibroblasts. This resulted in two attenuating amino acid substitutions in the E2 protein (32), one of which enhances binding to heparan sulfate (31).

Although our CHIKV strains also differed in the cell type of ultimate passage, this did not appear to have a major influence on virulence in the A129 model. The mosquito-derived isolates Asian/American HIII0044 and WA 37997 were ultimately passaged on Vero cells prior to A129 mouse infection and showed no difference in time-to-death from other strains in their respective lineages passaged ultimately on C6/36 mosquito cells. This suggested no major effect of virus generation in vertebrate versus mosquito cells. On the other hand, the two Asian strains 15561 and SV-0444-95 did kill A129 mice at significantly different rates, despite both being isolated from human serum and passaged on Vero cells immediately prior to infection. These results suggest minor intraclade virulence variation, which could reflect minor differences in passage histories but more likely results from minor genetic variation.

The CHIKV YO123223 strain that we used to challenge cynomolgus macaques differed considerably in passage history from the LR strain used in the past NHP study. While YO123223 was isolated from human serum prior to passaging twice on C6/36 cells and twice on Vero cells, the LR human isolate was rescued from an infectious cDNA clone (22) generated from viral RNA after 5 passages in Vero cells, one in suckling mice, and one in C6/36 mosquito cells (38). While the passage history of LR virus could contribute to its virulence in macaques, the lack of significant differences in murine virulence between LR and the other IOL CHIKV strains that we used suggests otherwise. Thus, although we cannot rule out the possibility that the absence of type I interferon signaling in the A129 model masks intraclade variation in strain virulence, our results suggest that LR has a representative, wild-type virulence phenotype.

Importantly, despite the pronounced variation in CHIKV virulence that we observed in both murine and NHP models, the CHIKV/IRESv1 vaccine candidate provided cross-lineage protection, preventing disease caused by challenge with an Asian/American lineage isolate despite the vaccine strain’s divergent IOL backbone. Further, the vaccine protected mice against challenge with a WA lineage strain 8 weeks after initial vaccination, indicating that not only is the vaccine cross-protective but it provides cross-protection for an extended period. Cross-protection was demonstrated not only in the A129 mouse model, where all vaccine doses prevented both morbidity and mortality, but also in the cynomolgus macaque model, where vaccination prevented interruption of normal diurnal temperature fluctuations and reduced or prevented viremia. However, there was a dose-dependent effect on immunogenicity, with doses of 10^4^ PFU or higher producing seroconversion in the majority of the macaques; all animals vaccinated with the lowest dose, 5 × 10^3^ PFU, failed to seroconvert postvaccination. However, after challenge with CHIKV strain YO123223, they developed only mild and short-lived viremia and did not exhibit signs of disease. This is an interesting phenomenon that suggests that a strong sustained antibody response is not necessarily required to control alphavirus infection; this observation is corroborated by past studies evaluating the efficacy of the Eilat virus/CHIKV chimeric vaccine. The killed version of this vaccine protected mice from lethal challenge, despite antibody titers measuring below 1:20 immediately before challenge for nearly half of the mice (39). The ability of the CHIKV/IRESv1 vaccine to cross-protect against heterologous CHIKV challenge in both mice and NHPs suggests that it may protect against all CHIKV strains; additional studies are needed to confirm this prediction, especially with the most divergent WA strains.

Finally, it has been suggested that Asian lineage CHIKV strains circulating in Philippines, which are close relatives of Asian/American strains, cause less-severe disease in humans (40). Although further clinical studies in more diverse locations are needed, our large-scale, animal model-based comparison of the virulence among all CHIKV lineages supports the hypothesis that Asian lineage strains, and especially members of the Asian/American sublineage, are less virulent than other CHIKV strains.

## MATERIALS AND METHODS

### Mice.

Four- to 6-week-old *IFNAR*^*−/−*^ A129 mice were obtained from a colony maintained at the University of Texas Medical Branch (UTMB, Galveston, TX) under pathogen-free conditions. Animals were monitored daily for signs of disease, including ruffled fur, hunched posture, lethargy, signs of dehydration, and significant weight loss. Any animal found moribund (significant lethargy, tremors, dehydration, and/or 20% weight loss) was humanely euthanized by CO_2_ inhalation. Mouse experiments were performed in an animal biosafety level 3 facility according to approved IACUC protocol 0608096B.

### Nonhuman primates.

Cynomolgus macaques were obtained from a commercial source (SNBL USA Ltd., Alice, TX), transported to the Tulane National Primate Research Center (TNPRC), and quarantined for 90 days. All experiments using cynomolgus macaques were approved by the Tulane Institutional Animal Care and Use Committee. The TNPRC is an Association for Assessment and Accreditation of Laboratory Animal Care International-accredited facility (AAALAC no. 000594). The U.S. National Institutes of Health (NIH) Office of Laboratory Animal Welfare assurance number for the TNPRC is A3071-01. Nonhuman primate housing consisted of individual open metal caging units that allowed visual recognition and prevented contact with other study animals in the room. Animals were maintained on standard primate chow supplemented daily with fresh fruit and vegetables. Animals were provided standard environmental enrichment during this study, which included manipulable items in cages, perches, foraging/task-oriented feeding methods, and human interactions with caretakers and research staff. All clinical procedures, including administration of anesthesia and analgesics, were carried out under the direction of a laboratory animal veterinarian. Animals were anesthetized with ketamine hydrochloride for blood collection procedures. Animals were preanesthetized with ketamine hydrochloride, acepromazine, and glycopyrolate; intubated; and maintained on a mixture of isoflurane and oxygen for telemetric implantation surgeries. Buprenorphine was given intraoperatively and postoperatively for analgesia. All possible measures were taken to minimize discomfort of all the animals used in this study. Appropriate medical care was implemented if any of these signs of illness were noted. If euthanasia was required in the judgment of the TNPRC veterinary staff, animals were euthanized in accordance with the recommendations of the Panel on Euthanasia of the American Veterinary Medical Association. The standard method of euthanasia for nonhuman primates at the TNPRC is anesthesia with ketamine hydrochloride (10 mg/kg of body weight) followed by an overdose of sodium pentobarbital. Tulane University complies with NIH policy on animal welfare, the Animal Welfare Act, and all other applicable federal, state, and local laws.

### CHIKV strains.

CHIKV strains 15561, LR 2006 OPY1, CHIKV/IRESv1 (vaccine strain), and CAR 256 were all rescued from infectious clones as previously described (38, 41, 42). CHIKV isolates TA0006 and HIII0044 were derived directly from patient and mosquito samples, respectively. All other CHIKV strains were kindly provided by Robert Tesh and Hilda Guzman of the World Reference Center for Emerging Viruses and Arboviruses (WRCEVA) at UTMB.

### Cell culture.

African green monkey (Vero) cells, human embryonic kidney (HEK-293) cells, and *Aedes albopictus* (C6/36) cells were obtained from the American Type Culture Collection, Manassas, VA. Vero cells were maintained in Dulbecco's modified Eagle medium (DMEM) supplemented with 1% penicillin-streptomycin (P/S) and 5% fetal bovine serum (FBS). HEK-293 cells were maintained in DMEM supplemented with 1% P/S, 10% FBS, and 1% nonessential amino acids and sodium pyruvate. C6/36 cells were maintained in DMEM supplemented with 10% FBS, 1% P/S, and 1% tryptose phosphate broth.

### Plaque assay.

Vero cells were grown to 90 to 100% confluence in either 12- or 6-well plates. Virus was diluted in DMEM supplemented with 5% FBS and from a series of 10-fold dilutions, 100 µl was plated per 12-well plate and 200 µl was plated per 6-well plate. After a 30-min incubation, an overlay composed of DMEM and 0.2% agarose was added and incubated for 24 to 48 h. Plates were then fixed with 10% formaldehyde for at least 1 h before staining with crystal violet, and plaques were counted.

### Plaque reduction neutralization.

Plaque reduction neutralization tests to determine PRNT_80_ titers were performed as previously described (43).

### Telemetry.

Subcutaneous radio telemetry transmitters combined with sensors capable of detecting biopotential signals of an electrocardiogram (ECG) as well as thermistor-type sensors capable of detecting temperature signals (T34G-8; Konigsberg Instruments [KI], Inc., Pasadena, CA) were surgically implanted under aseptic conditions. Following surgical implantation, animals were housed with cage-mounted antennae (TR38-1FG; Konigsberg Instruments, Inc.) configured to receive and transmit signals to a KI data acquisition base station. Data collection was continuous, and data were recorded for periods of 48 to 72 h using the Notocord-hem acquisition system (Notocord, Paris, France) and reported as hourly averages for 1-h observation intervals, with each animal serving as its own control. Baseline preexposure data averages were generated from each respective control subject for a minimum of 6 days. Preexposure data were aligned by the time of day over a 24-h period and averaged for hourly means to establish detection thresholds for each animal. These thresholds of detection of significant events were defined to be greater than 1.5 times the maximum standard deviation of the preexposure control averages. Individual postexposure data were then aligned by time and compared against preexposure values for each animal. An assessment of fever (hyperthermia) hours, hypothermia hours, and fever intensity was then performed for each animal.

### Statistics.

For lineage comparison studies in A129 mice, isolates were pooled into their respective lineages for all analyses. Kaplan-Meier survival curves were generated and analyzed in SigmaPlot (Systat, San Jose, CA). Viremia, weight, and footpad data were analyzed using one-way ANOVA using SPSS Statistics software (IBM Corp., Armonk, NY); one-way ANOVAs were employed rather than repeated measures to account for increasingly unequal representation among groups over time. Significant telemetry data changes among NHP vaccination groups were compared statistically using the standard *t* test.

### Ethics statement.

All animal work was performed in strict adherence to guidelines set forth by relevant governing institutions, including national and local agencies.
