# Supplementary figures and images for: Chikungunya Virus Strains Show Lineage-Specific Variations in Virulence and Cross-Protective Ability in Murine and Nonhuman Primate Models
Source: mBio. 2018 Mar 6;9(2):e02449-17. doi: 10.1128/mBio.02449-17 (PMC5844994; doi:10.1128/mBio.02449-17)

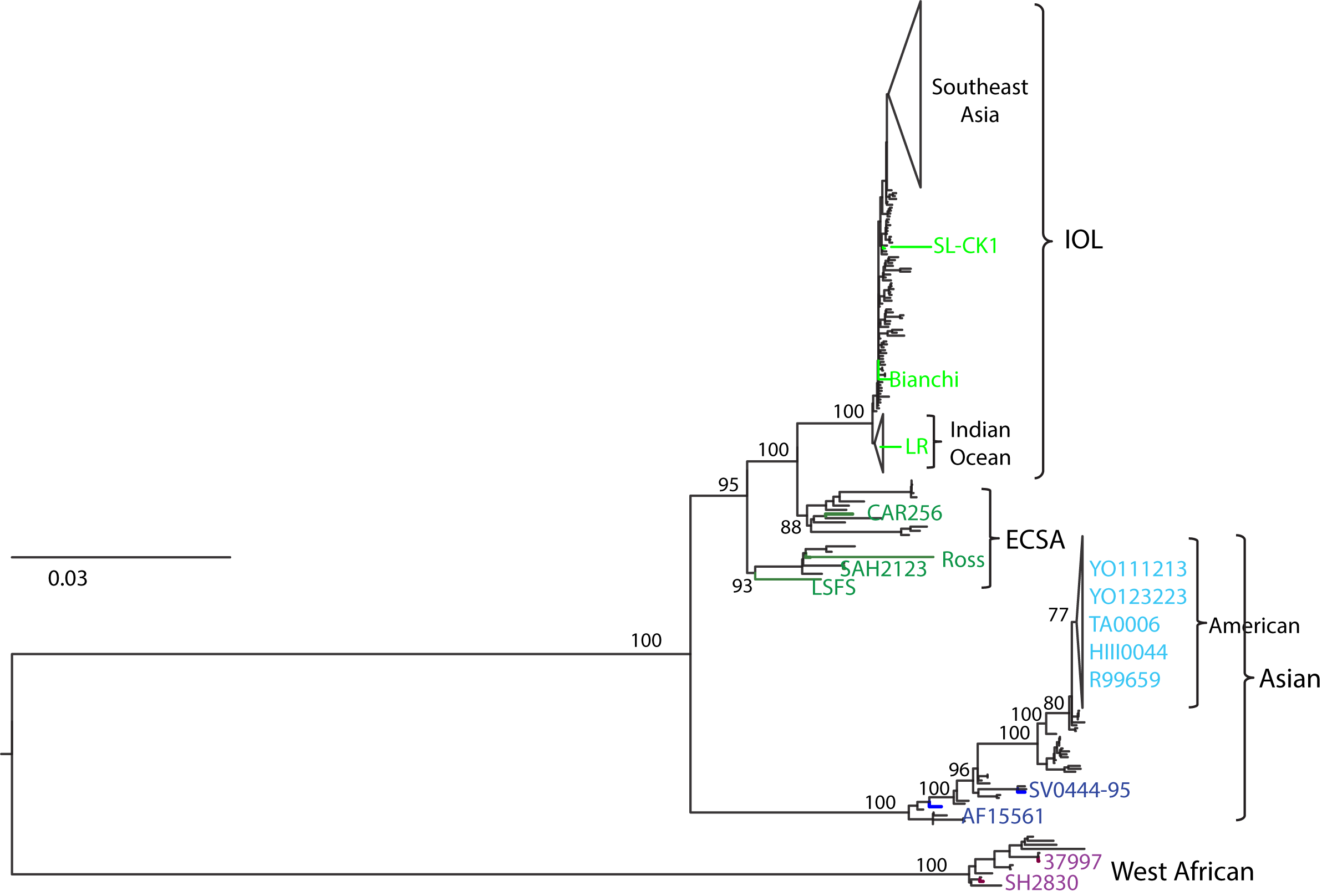

Supplement: FIG S1 [file mbo001183753sf1.tif]

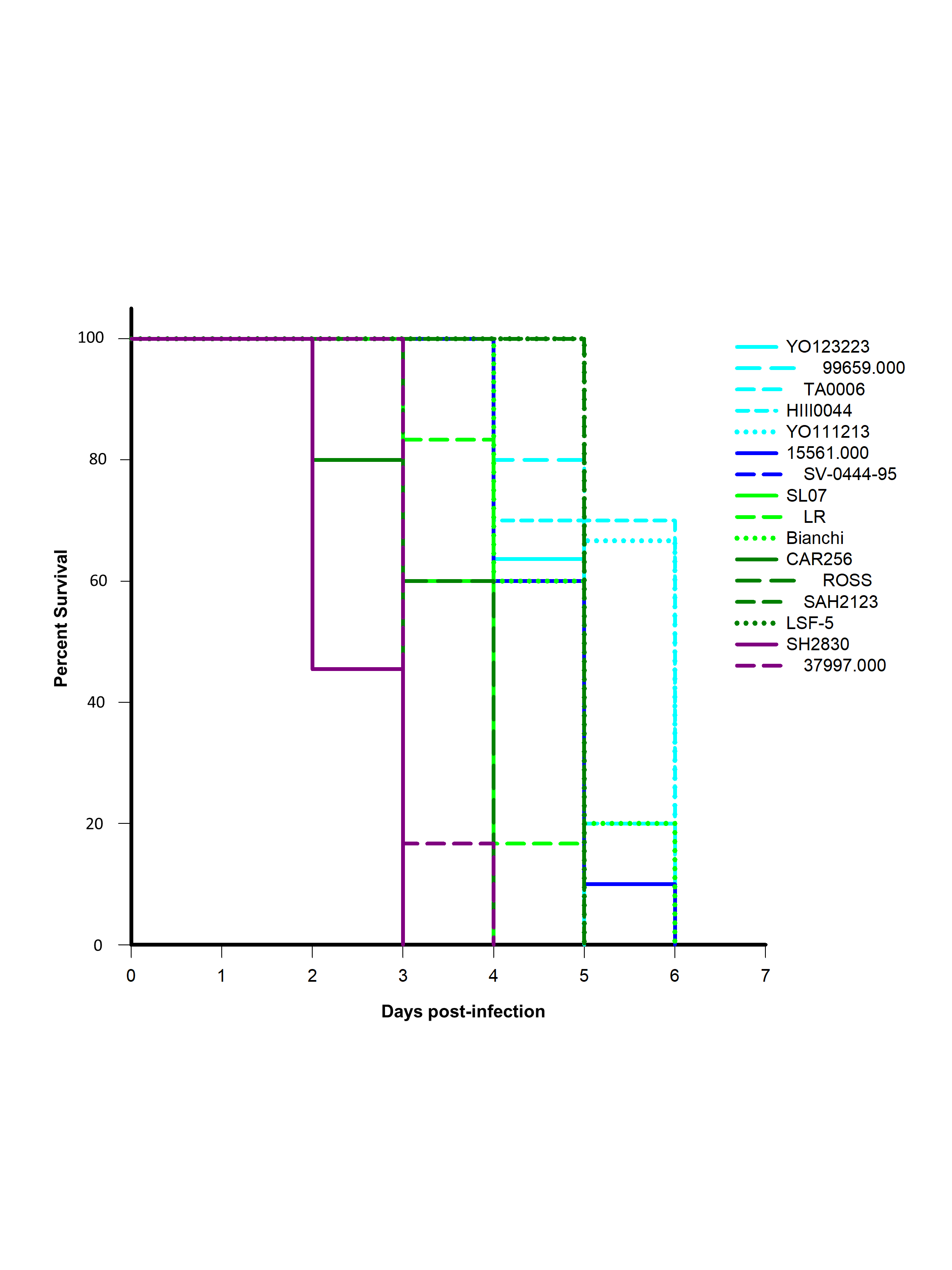

Supplement: FIG S2 [file mbo001183753sf2.tif]

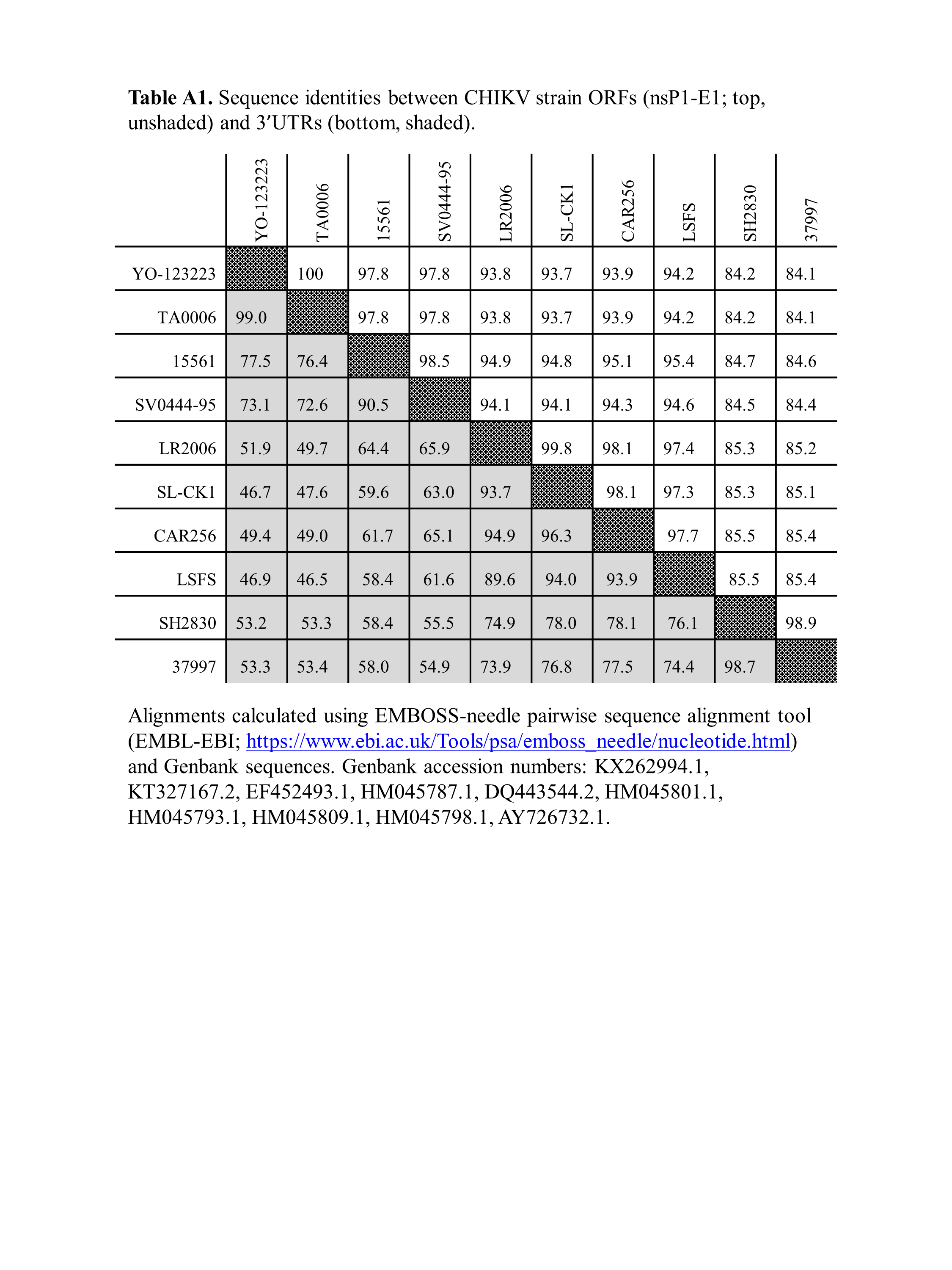

Supplement: TABLE S1 [file mbo001183753st1.tif]
